# Hypomethylation of *IL1RN* and *NFKB1* genes is linked to the dysbalance in IL1β/IL-1Ra axis in female patients with type 2 diabetes mellitus

**DOI:** 10.1371/journal.pone.0233737

**Published:** 2020-05-29

**Authors:** Sona Margaryan, Eva Kriegova, Regina Fillerova, Veronika Smotkova Kraiczova, Gayane Manukyan

**Affiliations:** 1 Department of Immunology, Faculty of Medicine and Dentistry, Palacký University and University Hospital Olomouc, Olomouc, Czech Republic; 2 Russian-Armenian (Slavonic) University, Yerevan, Armenia; 3 Institute of Molecular Biology, Laboratory of Molecular and Cellular Immunology, Yerevan, Armenia; East Tennessee State University, UNITED STATES

## Abstract

Inflammation has received considerable attention in the pathogenesis of type 2 diabetes mellitus (T2DM). Supporting this concept, enhanced expression of interleukin (IL)-1β and increased infiltration of macrophages are observed in pancreatic islets of patients with T2DM. Although IL-1 receptor antagonist (IL-1Ra) plays a major role in controlling of IL-1β-mediated inflammation, its counteraction effects and epigenetic alterations in T2DM are less studied. Thus, we aimed to analyze the DNA methylation status in *IL1RN*, *RELA (p65)* and *NFKB1 (p50)* genes in peripheral blood mononuclear cells (PBMCs) from treated T2DM patients (n = 35) and age-/sex- matched healthy controls (n = 31). Production of IL-1β and IL-1Ra was analyzed in plasma and supernatants from LPS-induced PBMCs. Immunomodulatory effects of IL-1β and IL-1Ra were studied on THP-1 cells. Average DNA methylation level of *IL1RN* and *NFKB1* gene promoters was significantly decreased in T2DM patients in comparison with healthy controls (*P*< 0.05), which was associated with the increased IL-1Ra (*P*< 0.001) and IL-1β (*P* = 0.039) plasma levels in T2DM patients. Negative association between average methylation of *IL1RN* gene and IL-1Ra plasma levels were observed in female T2DM patients. Methylation of *NFKB1* gene was negatively correlated with IL-1Ra levels in the patients and positively with IL-1β levels in female patients. LPS-stimulated PBMCs from female patients failed to raise IL-1β production, while the cells from healthy females increased IL-1β production in comparison with unstimulated cells (*P*< 0.001). Taken together, the findings suggest that hypomethylation of *IL1RN* and *NFKB1* gene promoters may promote the increased IL-1β/IL-1Ra production and regulate chronic inflammation in T2DM. Further studies are necessary to elucidate the causal direction of these associations and potential role of IL-1Ra in anti-inflammatory processes in treated patients with T2DM.

## Introduction

Type 2 diabetes mellitus (T2DM) is a chronic metabolic disorder characterized by hyperglycemia, loss of insulin sensitivity and progressive dysfunction of pancreatic β cells [1]. T2DM is strongly associated with age, obesity, and physical inactivity in subjects with a genetic predisposition [2]. Despite the etiology of T2DM is multifaceted, chronic subclinical inflammation that can be attributed to the dysregulation of innate immune system is believed to have a major role in pathogenesis of the disease. Multiple evidences support the presence of islet inflammation including observations of enhanced expression of pro-inflammatory interleukin (IL)-1β, various cytokines and chemokines, increased infiltration of CD68^+^ macrophages [3,4] which were shown to contribute to the local inflammation, insulin resistance and pancreatic dysfunction in T2DM [4,5].

Islet macrophages are the major contributors to the islet IL-1β secretion [6, 7]. Being powerful inflammation-promoting cytokine, its secretion and activity are kept under strict control. IL-1β activity is regulated by an endogenous inhibitor IL-1 receptor antagonist (IL-1Ra) [8]. Both members of IL-1 family binds with high affinity to specific IL-1 receptors type 1 (IL-1R1) leading to intracellular signal transduction and, as a result, cellular responses [9]. IL-1β and IL-Ra are regulated at the transcriptional level through nuclear factor-κB (NF-κB) expression, and IL-1β additionally through inflammasome formation [9–11]. Monocytes/macrophages are the major source of IL-1β and IL-1Ra during inflammation producing these cytokines in an auto-regulatory feedback loop [12]. Pancreatic β cells have the most abundant expression of IL-1R1 compared to other cells and tissues [13]. Thus, the fine-tuned balance between IL-1β and IL-1Ra at the IL-1R1 site is critical in determining responses in pancreatic β cells and finally to the progression of the diseases in general.

A number of features of T2DM have been proposed to be due to epigenetic effects, including DNA methylation which might represent important link between genetic and environmental factors in the development of T2DM [14]. In particular, epigenetic regulation plays a marked role in controlling macrophage function in T2DM [15]. Macrophages exhibit marked plasticity and are able to rapidly adjust their phenotype and transcriptional program in response to a wide range of environmental signals via the changes in epigenetic landscape [16]. Thus, epigenetic changes in monocyte/macrophage programming may predict a scenario of pathophysiological events and disease risk. In the present study, we questioned whether DNA methylation status of *IL1RN*, *RELA (p65)* and *NFKB1 (p50)* genes may be relevant to IL-1β and IL-1Ra production and may be a possible contributory factor in the inflammatory pathogenesis of T2DM. The study is also aimed to define immunomodulatory effects of IL-1β and IL-1Ra on THP-1 cells, in the concentrations corresponding to the levels in the blood of T2DM patients.

## Material and methods

### Subjects

A total of 35 patients with T2DM (mean age 53.78 ± 6.4) and 31 healthy controls (mean age 51.9 ± 7.6) were enrolled in this study. Clinical characteristics of T2DM patients are shown in Table 1. Blood samples were received from “Surb Astvatsamayr” Medical Center (Yerevan, Armenia). Inclusion criteria for T2DM patients were: 1) fasting plasma glucose concentration ≥7.0mmol/L; 2) random venous plasma glucose concentration ≥11.1mmol/L; 3) glycosylated hemoglobin levels HbA_1_ >48mmol/mol (6.5%). In parallel with nutrition therapy recommendations, all diabetic patients were receiving gliclazide and metformin containing hypoglycemic medications. T2DM patients with long-term complications or concomitant disorders were excluded. Healthy controls were age- and sex-matched, had no family history of diabetes/obesity or other metabolic disorders, with normal random venous and fasting glucose levels. The study was approved by the Ethical Committee of the Institute of Molecular Biology of the National Academy of Sciences of the Republic of Armenia (IRB IORG0003427) and written informed consent was obtained from all participants.

**Table 1:** Clinical characteristics of T2DM subjects and healthy individuals.

	Healthy (n = 31)	T2DM (n = 35)
Age (min-max)	51.9 (40–67)	53.78 (46–69)
Gender (male/female)	16/15	15/20
BMI (kg/m^2^)	23.51 ± 2.61*	25.7 ± 3.51
Fasting glucose (mmol/L)	4.8 ± 0.36	8.21 ± 2.4
HbA1c (%)	5.1 ± 0.6	7.8 ± 1.2
LDL (mmol/L)	2.3 ± 0.47	2.7 ± 0.33
HDL (mmol/L)	1.5 ± 0.2	1.2 ± 0.4
Treatment Metformin/combination of metformin and gliclazide	-	9/26

*Results are represented as mean ± SD.

### DNA methylation assay in PBMCs

Methylation levels of *IL1RN*, *RELA* and *NFKB1* gene promoter regions were determined in peripheral blood mononuclear cells (PBMCs) isolated by density gradient centrifugation using Histopaque-1077®. DNA was extracted from PBMCs using the salt out method [17].

EpiTYPER® technology [18] was used to quantitatively assess DNA methylation of multiple CpGs in promotor regions of studied genes. Assay setup and validation for each primer-pair was performed using 100%, 50% and 0% methylated control DNA samples (EpiTect Control DNA, Qiagen). Bisulfite treatment of genomic DNA was performed with the EZ DNA Methylation Kit^™^ (Zymo Research, Irvine, CA, USA), according to manufacturer's protocol. PCR reactions, SAP treatment and T cleavage were carried out using MassCleave kit (Agena Bioscience, San Diego, USA) accoding the manufacturer instructions. Briefly, primers (designed by EpiDesigner (Agena Bioscience)) tagged with T7 promoter were applied for PCR reactions with bisulfite converted DNA (20 ng/μL for each well). For PCR following thermal conditions were applied: 4 minute at 94 °C, then 20 seconds at 94°C, 30 seconds at 56°C, 1 minute at 72 °C_ repeated 45 times, then 3 minutes at 72°C. After PCR, shrimp alkaline phosphatase treatment was done to remove unincorporated nucleotides. For the final step, amplified samples were treated with T7 polymerase and incubated for 3 hours at 37°C. Methylation degree of CpG sites in target genes was assessed using the MassArray® (Agena Bioscience) and results were analyzed with Epityper 1.4 software.

### PBMC culturing

Briefly, isolated PBMCs (1x10^6^ cells/mL) were cultured in RPMI-1640 containing 2g/L of D-glucose and supplemented with 10% fetal bovine serum and 2 mM L-Glutamine in absence or presence of LPS (100 ng/mL) for 24 hours at 37°C in 5% CO_2_ environment. After LPS stimulation, supernatants were harvested and stored at -80°C until determination of IL-1β and IL-1Ra levels.

### THP-1 cell line culturing

Human THP-1 monocytic cells (ECACC #88081201) were cultured in RPMI-1640 medium supplemented with 10% heat-inactivated FBS, 2 mM L-Glutamine, 10 mM HEPES, penicillin (100 U/mL), streptomycin (100 mg/L) as recommended by the manufacturer. Corresponding concentration of mannitol was added to the high glucose containing media in order to achieve osmotic balance. Cells between passages 4 and 8 were used in the experiments.

THP-1 cells were differentiated into M0 macrophages by PMA (50 ng/mL) treatment for 72 hours in low glucose (L-Glu, 5 mM) and high glucose (H-Glu, 25 mM) conditions, respectively. Differentiated cells were washed and maintained in L-Glu/H-Glu media for 6 hours and stimulated with different concentrations of human recombinant IL-1β (2.5 ng/mL) and IL-1Ra (250 and 500 ng/mL) (Biolegend, USA). The cells were harvested using trypsin-EDTA solution and used for determination of phagocytic activity and immunophenotyping. Culture supernatants were collected and frozen at -80°C for TNF-α and IL-10 measurement.

### Phagocytosis assay

For the assessment of phagocytosis, before adding pHrodo^™^ Green Zymosan BioParticles® (ThermoFisher Scientific, MA, USA) THP-1 cells were pre-stimulated with LPS (100 ng/mL) for 2 hours at 37°C in 5% CO_2_ atmosphere. Further, labeled cells were analyzed using flow cytometry.

### Flow cytometry analysis

Optimal concentrations of monoclonal antibody combinations directed against the following human surface antigens: HLA-DR-FITC, CD181-FITC (CXCR1), CD86-PE, CD282-PE (TLR2), CD80-PerCP/Cy5.5, CD11b-PerCP/Cy5.5, CD16-PE/Cy7, CD163-PE/Cy7, CD197-APC (CCR7), CD284-APC (TLR4), CD206-APC/Cy7, CD64-BV421, CD14-BV510 (BioLegend) were incubated with THP-1 cells stimulated with IL-1β/IL-1Ra. Isotype matched FITC, PE, PerCP/Cy5.5, PE/Cy7, APC and APC/Cy-7-conjugated irrelevant antibodies (BioLegend) were used as negative controls. Antigen expression was analyzed on a BD FACSCanto II cytometer (BD Biosciences, San Jose, CA). Obtained data were analyzed using FlowJo V10 software (FlowJo, Ashland, OR, USA). In all experiments, a minimum of 10,000 events were counted. Results were expressed as a percentage and median fluorescence intensity (MFI) of the cells for each examined marker.

### Production of IL-1β, IL-1Ra, IL-10 and TNF-α

Concentrations of IL-1β and IL-1Ra in plasma samples and supernatants of cultured PBMCs were detected using ELISA MAX Deluxe kit (Biolegend, USA) and SimpleStepELISA® kit (Abcam, UK), respectively. The levels of IL-10 and TNF-α in supernatants from THP-1 cells were measured with ELISA MAX Deluxe kit (Biolegend, USA).

### Statistical analysis

Statistical analyses were carried out using GraphPad prism version 5.0 (San Diego, CA). Results are presented as mean ± SEM. Parametric and non-parametric T tests (based on data distribution), one way repeated measure ANOVA, were applied for data analysis. Spearman and Pearson correlation analyses were performed to determine the association between variables. Statistical significance was defined as *P*≤ 0.05.

## Results

### Patterns of IL1RN, RELA and NFKB1 gene methylation in PBMCs

Methylation status of *IL1RN*, *RELA (p65)*, and *NFKB1 (p50)* gene promoter was determined in 35 T2DM patients and 31 healthy individuals.

**Fig 1:**
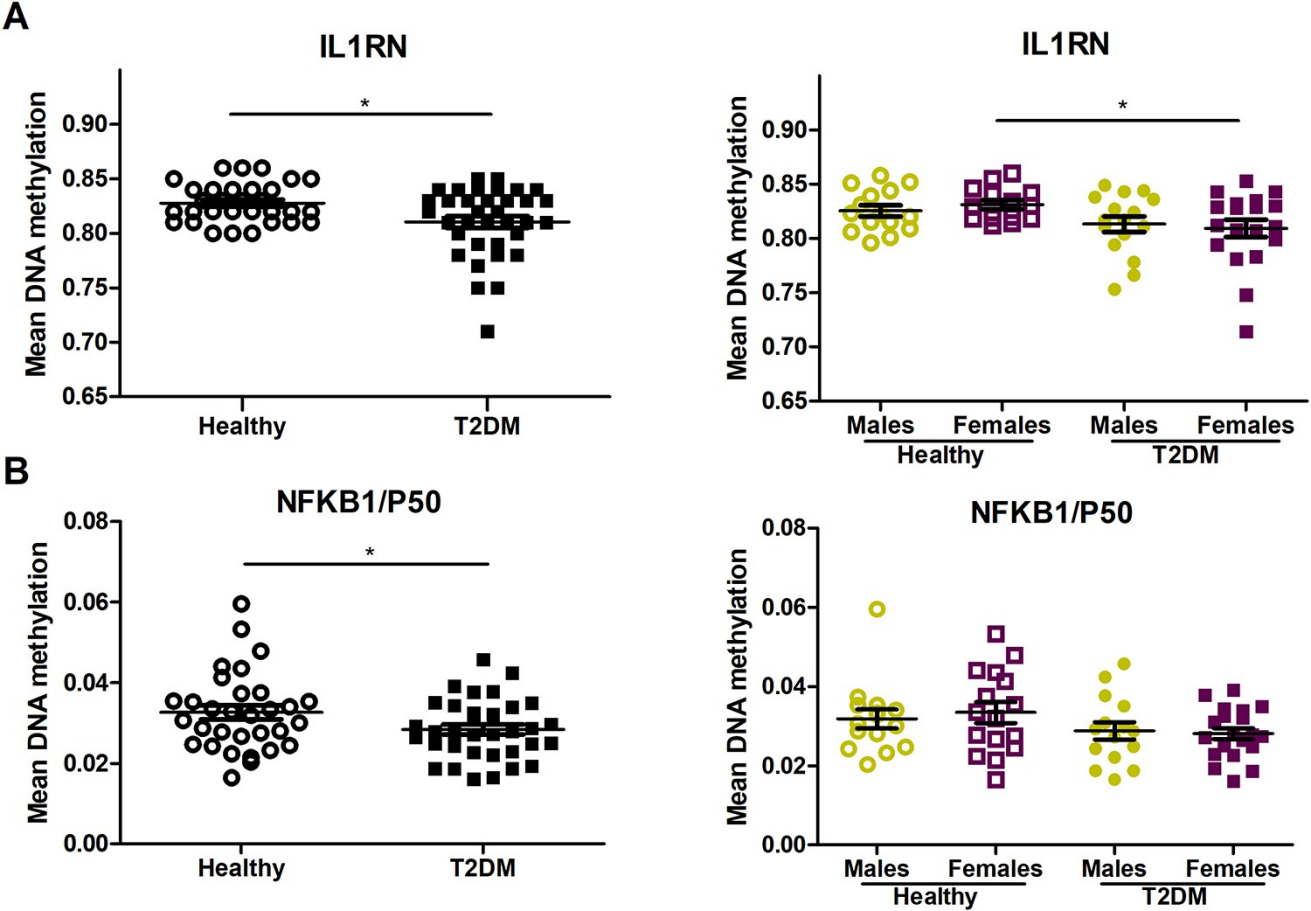
Average DNA methylation levels of target genes in PBMCs from type 2 diabetes mellitus (T2DM, n = 35) patients and healthy controls (Healthy, n = 31). (A) Mean methylation levels in *IL1RN* gene promoter (across 16 CpG loci) in all subjects and males/females; (B) Mean methylation levels in *NFKB1* gene promoter (across 65 CpG loci) in all subjects and males/females. Results are expressed as mean ± SEM. *P< 0.05.

Analyses of methylation data revealed that an average methylation value of *IL1RN* gene promoter was reduced in diabetic group compared with healthy one (*P* = 0.043). Significant differences in average methylation levels were caused predominantly by the differences between female patients and female healthy controls (*P* = 0.047) (Fig 1A). Among 16 CpG loci (all CpGs in the promotor) of *IL1RN* gene promoter, methylation levels of two CpGs (chromosome 2; CpG 302, 383) were significantly decreased (*P*< 0.05) in T2DM patients compared with healthy subjects (S1 Table). These two hypomethylated islands within *IL1RN* promoter are located in the region containing regulatory elements for *IL1RN* gene (GeneHancer GH02J113113), including binding sites for several transcription factors, but not NF-κB.

NF-κB binds to DNA as a dimer formed by the combination of proteins including subunit p65 (RelA) and subunit p50 (NF-κB1) [19]. Methylation status of 100 CpG (52% of the whole promoter sequence) loci in promoter region of *NFKB1* gene was analyzed. Methylation levels in 35 of 100 CpG loci were extremely low or undetectable in patients and controls. In the rest 65 CpG sites, the overall methylation status was decreased in T2DM patients compared to healthy group (*P* = 0.047) (Fig 1B).

57 CpG sites (49% of the whole promoter sequence) in *RELA* gene promoter region were analyzed. The majority of CpG loci in *RELA* gene were demethylated in the cells from both diseased and healthy groups. No association between *IL1RN*, *RELA* and *NFKB1* gene promoter methylation levels was found in both studied groups.

### IL-1β and IL-1Ra plasma levels are increased in T2DM patients

**Fig 2:**
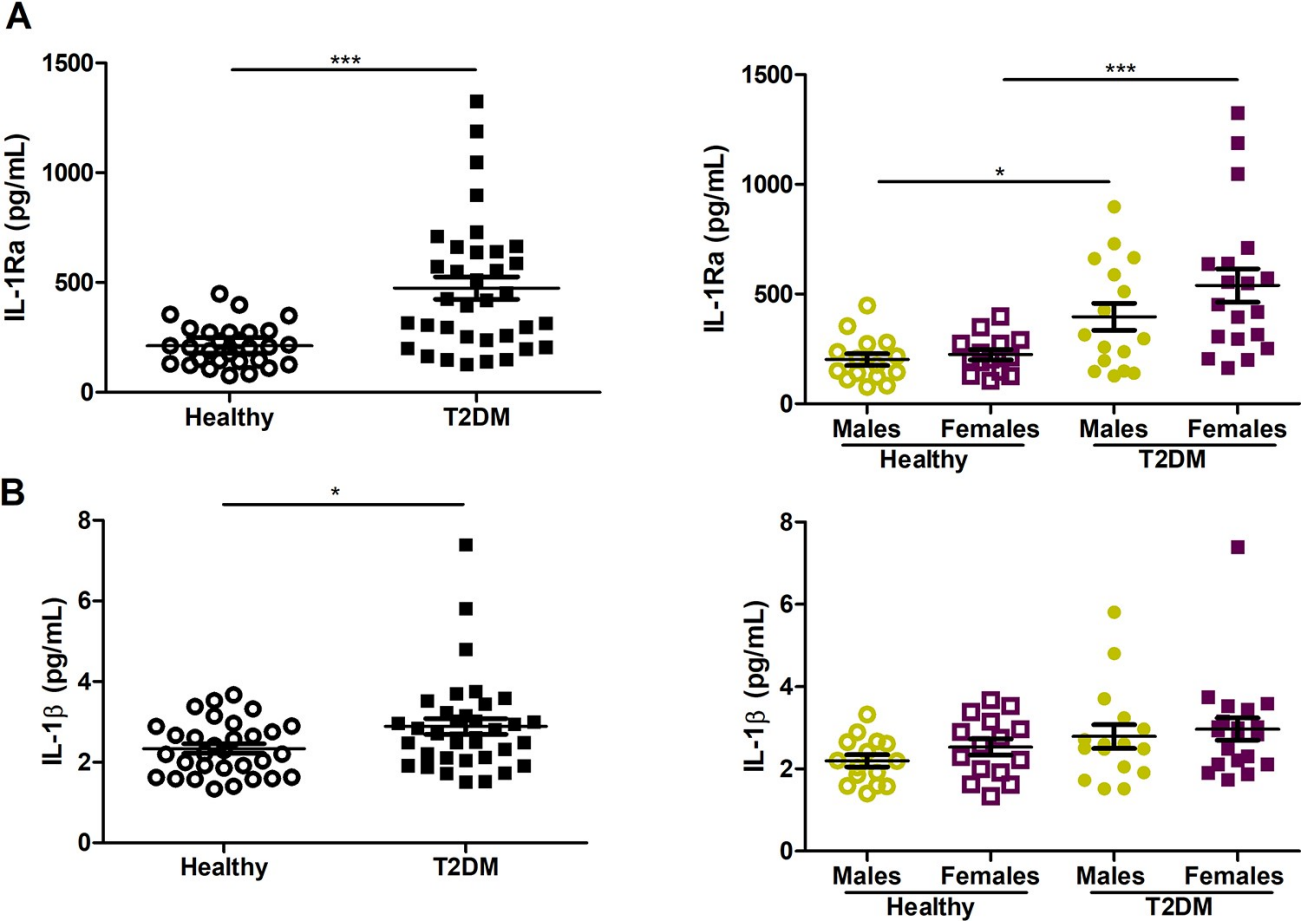
Plasma levels of cytokines in type 2 diabetes mellitus (T2DM, n = 35) patients and healthy controls (Healthy, n = 30). (A) Levels of IL-1Ra in all subjects and males/females; (B) Levels of IL-1β in all subjects and males/females. Results are expressed as mean ± SEM. *P< 0.05, ***P< 0.001.

Next, we explored whether DNA methylation status in T2DM patients is associated with the phenotypic discordance. IL-1Ra plasma levels were remarkably increased in the patients compared with controls (2.5-fold, *P*< 0.001). The differences in IL-1Ra plasma levels were more prominent in female patients (*P*< 0.001) than in males (*P* = 0.019) (Fig 2A). Similarly, plasma of T2DM patients was characterized by slightly increased IL-1β levels (*P* = 0.038). Significant differences between females and males in both groups were not found (Fig 2B). IL-1Ra/IL-1β ratio was found to be increased in diseased group compared to healthy one (*P* = 0.001) and in female patients compared to healthy females (*P* = 0.001) (S1 Fig).

Notably, plasma levels of two opposite-acting cytokines IL-1β and IL-1Ra were positively correlated (*r* = 0.43, *P* = 0.019) in healthy group (S2A Fig), while any association between these two cytokines in T2DM patients was not observed even when female and male patients were analyzed separately.

### Decreased methylation of IL1RN is associated with increased IL-1Ra plasma levels in female T2DM patients

**Fig 3:**
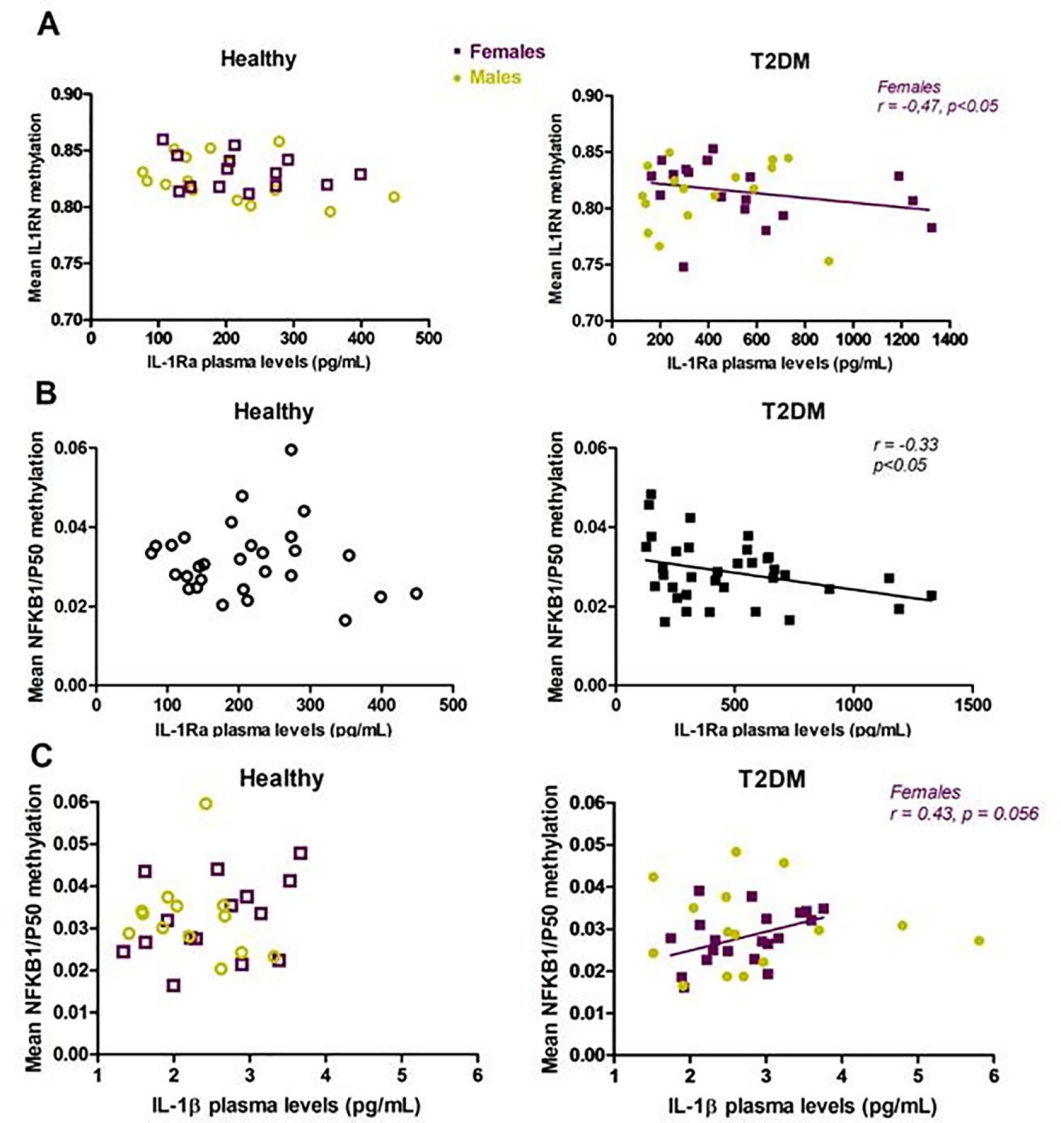
An association between mean DNA methylation and cytokine plasma levels in type 2 diabetes mellitus (T2DM) patients and healthy controls (Healthy). (A) Correlation between *IL1RN* promoter mean methylation level and IL-1Ra plasma levels in males (green) and females (violet); (B) Correlation between *NFKB1* promoter mean methylation level and IL-1Ra plasma levels in all studied subjects (black); (C) Correlation between *NFKB1* promoter mean methylation level and IL-1β plasma levels in males/females.

In T2DM patients, an association between average methylation levels of *IL1RN* gene promoter and plasma IL-1Ra levels was not observed. Nevertheless, there was a negative correlation between mean methylation status of *IL1RN* and IL-1Ra plasma concentration in female patients (*r* = -0.47, *P* = 0.047) (Fig 3A). Similarly, a negative association between 2 CpG positions and IL-1Ra levels was observed only in female patients: CpG 383 (*r* = -0.47, *P* = 0.037) and CpG 232 (*r* = -0.489, *P* = 0.028). In healthy controls, any correlation between average methylation levels of *IL1RN* and IL-1Ra plasma levels was not found (Fig 3A). A negative correlation was found between two CpG loci of *IL1RN* gene (chromosome 2; CpG loci 343, 383) and IL-1Ra plasma levels in healthy individuals (*r* = -0.40, *P* = 0.031 and *r* = -0.38, *P* = 0.040, respectively). The plasma levels of IL-1β were not correlated with *IL1RN* epigenetic modifications both in healthy controls and patients.

In healthy controls, the overall *IL1RN* promoter methylation level was decreasing along with the age (*r =* -0.44, *P =* 0.014). This association was found to be in consistency with a positive correlation between high IL-1Ra plasma concentrations and age (*r =* 0.46, *P =* 0.018) (S2B Fig). In diseased group, an association of *IL1RN* promoter methylation levels with age and clinical parameters, such as fasting glucose and total cholesterol levels, was not found.

### Methylation levels NFKB1 gene correlate with IL-1β plasma levels in female patients

To determine up-stream regulation of IL-1β/IL-1Ra axis, we have analyzed an association of *NFKB1* gene methylation with plasma levels of these two cytokines. Interestingly, *NFKB1* gene methylation status was correlated only with IL-1β and IL-1Ra levels in T2DM patients: negatively with IL-1Ra levels (*r* = -0.33, *P* = 0.049) and positively with IL-1β levels (*r* = 0.43, *P* = 0.056) in the plasma of female patients (Fig 3B and 3C).

### LPS-induced production of IL-1β and IL-1Ra by PBMCs

**Fig 4:**
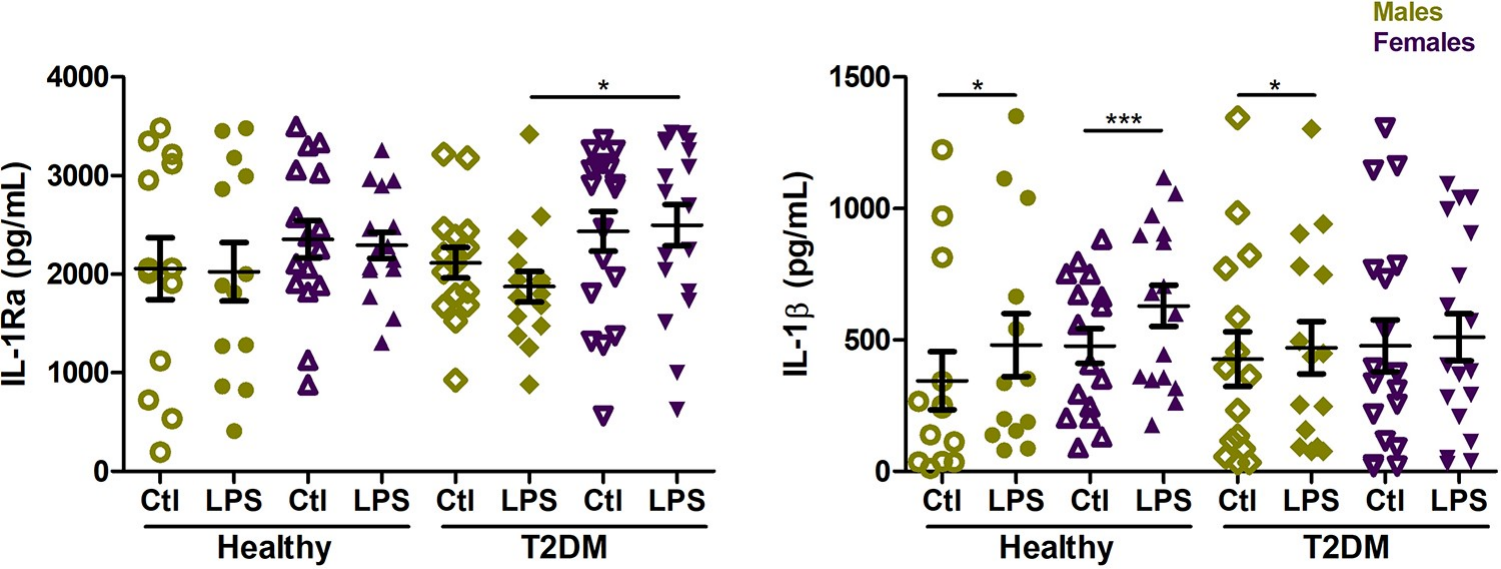
LPS-stimulated production of IL-1Ra and IL-1β by PBMCs from type 2 diabetes mellitus patients (T2DM, n = 32) and healthy controls (Healthy, n = 27). PBMCs isolated from males (green) and females (violet) were treated with LPS (100ng/ml) and left unstimulated as a control (Ctl) for 24h. Data are presented as mean ± SEM. *P< 0.05, ***P< 0.001.

To estimate the ability of PBMCs from both studied groups to respond to bacterial stimuli, we have induced the cells with LPS and analyzed production of IL-1β and IL-1Ra in supernatants. There were no significant differences in IL-1β and IL-1Ra levels in response to LPS stimulation between studied diseased and healthy groups. Nevertheless, LPS exposure resulted in increased release of IL-1β compared to unstimulated samples except female patients. Particularly, LPS induced IL-1β production by PBMCs from healthy and diseased males (*P* = 0.011 and *P* = 0.032, respectively) and in healthy females (*P*< 0.001). IL-1Ra production in response to LPS was higher in female T2DM patients in comparison with male patients (*P* = 0.027) (Fig 4).

Any association between IL-1β and IL-1Ra plasma levels and LPS-stimulated production of these cytokines by PBMCs was not observed. However, correlation analysis of clinical parameters and LPS-stimulated production of IL-1β/IL-1Ra revealed a positive correlation between fasting glucose levels and IL-1Ra production in the patients (*r* = 0.38, *P* = 0.031) (S3 Fig).

### Differential action of IL-1β and IL-1Ra on THP-1 cells

To analyze functional and immunophenotypic consequences of the increased IL-1Ra levels in the context of hyperglycemic conditions, we have used recombinant protein IL-1Ra in the concentrations corresponding to the levels found in the plasma of patients and healthy controls in L-Glu and H-Glu conditions (fold change between IL-1Ra and IL-1β plasma levels was 100x and 200x in healthy controls and T2DM patients, respectively).

First, we have assessed potential of IL-1β and IL-1Ra to shift M1 and M2 macrophage polarization. The influence of IL-1β and IL-1Ra stimuli on polarization ability of M0 macrophages was not observed neither in L-Glu nor in H-Glu conditions. IL-1β and IL-1Ra stimuli have led to the combined expression of both M1 (CD86, CD80, CCR7, HLA-DR) and M2 (CD163, CD206) polarization markers.

Next, we analyzed differences in expression of inflammation-related markers (CD14, CD16, CD64, CD11b, TLR2, TLR4, CXCR1). Only CXCR1 marker exhibited significant changes in our experimental model. The highest expression of CXCR1 was observed in H-Glu media with the concentrations of IL-1β and IL-1Ra found in the patient’s plasma (*P* = 0.009). The up-regulation seems to be caused by the increased levels of IL-1Ra (500 ng/mL), not high glucose content, since CXCR1 expression levels in L-Glu and H-Glu media at the same concentrations of IL-1Ra (250 ng/mL) were not different (S4A Fig).

In the next step, we examined phagocytic activity of THP-1 cells using pHrodo^™^ fluorescent bioparticles. In L-Glu, IL-1β and IL-1Ra stimulation reduced particle uptake in THP-1 cells (*P =* 0.054), while in H-Glu group the same combination of cytokines slightly enhanced particle uptake. There was a tendency for a decreased uptake of pHrodo bioparticles in THP-1 cells in H-Glu condition compared to L-Glu one. Besides, reduced phagocytic activity was observed in diabetes-associated concentrations of cytokines (200x difference) compared to physiological concentrations in healthy subjects (100x, *P =* 0.007) (S4B Fig).

### IL-10 and TNF-α production

Production of IL-10 in L-Glu group was reduced after IL-1β and IL-1Ra induction (100x), however this difference did not reach statistical significance (*P* = 0.093, S4C Fig). While in H-Glu group the same combination of IL-1β and IL-1Ra slightly increased IL-10 production. Production of TNF-α was slightly up-regulated in H-Glu group, while any difference after IL-1β and IL-1Ra stimulation was not observed in both groups (S4D Fig).

## Discussion

Extensive clinical and experimental studies suggest that IL-1Ra regulates development and progression of a variety of chronic inflammatory diseases [20] including T2DM [21]. Molecular mechanisms responsible for controlling endogenous production of IL-1Ra by monocytes are currently largely unknown. Our study for the first time showed hypomethylation of *IL1RN* and *NFKB1* promotor regions in PBMCs of patients with T2DM compared with healthy subjects. Our results are in line with the concept that decreased DNA methylation is a feature of transcriptionally active genes and may contribute to the enhanced production of IL-1Ra as seen in our patients (S5 Fig). Hypomethylation of *IL1RN* gene as well as correlation between average *IL1RN* methylation and IL-1Ra plasma levels were observed only in female patients. In line with our observation, markedly higher serum levels of IL-1Ra in women with T2DM, even after BMI correction, have been reported [22]. Similarly, the capacity of the cells to secrete IL-1Ra in response to LPS was also increased in female patients compared to male patients. The ability of the cells to produce increased concentrations of IL-1Ra might be caused by the effect of estrogens which decrease fasting glucose while impairing glucose tolerance delaying the onset of type 2 diabetes in women [23], characteristics linked to the lower prevalence of T2DM in women [24].

An increased plasma IL-1β and IL-1Ra protein levels in T2DM subjects were not replicated entirely by the LPS-stimulated production of these cytokines. The discrepancy may be explained by the refractory state of monocytes from the patients as evidenced by the failure of the cells from female patients to increase IL-1β production in response to LPS. Low-grade increase in plasma LPS termed “metabolic endotoxemia” is a well-known feature of T2DM [25–27]. “Metabolic endotoxemia” is able to program innate immune responses [28] and contribute to chronic low-grade inflammation [25, 29, 30]. This chronic stimulus by the altered metabolic state in T2DM could promote hyporesponsiveness or so called tolerant state of the cells with a minor release of cytokines which was shown previously on human monocytes [31] and rat neutrophils [32, 33]. Additional mechanisms such as epigenetics may play a role in memory-like activity in innate immune cells and endotoxin tolerance [34, 35]. It was shown that LPS tolerance disrupts the activating stage of NF-κB p65 and altered nucleosome remodeling at the IL-1β promoter in THP-1 cells [36]. DNA methylation have not been widely studied regarding to LPS tolerance and deserves further studies.

Increased IL-1β and IL-1Ra plasma levels might be caused by genetic and non-genetic factors [37]. In T2DM, exposure of pancreatic islets to glucose, leptin, and free fatty acids induces the production of IL-1β and contribute to β-cell failure [38, 39]. IL-1β stimulates its own production and IL-8 release by β-cells and attracts macrophages which in turn produce IL-1β and other cytokines [40, 41]. In present study, we showed that levels of IL-1β and IL-1Ra in the concentrations corresponding to the levels found in the plasma of the patients under H-Glu conditions up-regulated expression of CXCR1 on THP-1 cells, which has been shown to drive macrophage infiltration in β-cells [41].

Interestingly, we have found a negative correlation between the levels of *NFKB1* gene methylation and the levels of IL-Ra in plasma of T2DM patients and positive correlation with IL-1β levels. NF-κB1 (p50) is one of the five members of the NF-κB transcription factor family which is IκB kinase-like protein [42, 43]. p50 can function either as a dimer partner for the p65 and c‐Rel or as a p50 homodimer. p65/c-Rel heterodimers induce gene expression, while p50 homodimers are generally transcriptional repressors modifying expression of NF-κB target genes, including those involved in inflammation [44]. Non-detectable methylation levels of p65 may count for a high activity of the subunit, however a degree of its cooperation with p50 in this context is difficult to predict. p50 homodimer overexpression and predominance of p50-p50 homodimers was shown to suppress IL-1β activation [45] and alter the expression of NF-κB-inducible genes, which was shown to lead to macrophage (M)2-like phenotype with high production of IL-10 [46].

In T2DM patients, there is controversial data on global methylation status showing both global hypo- [47] and hyper-methylation of CpG islands [48, 49]. Moreover, it has been shown that treatment of diabetic patients resulted in reversal of aberrant DNA methylation patterns and has led to the normalization of DNA methylation profile as in healthy controls [49], suggesting that changes in methylation profile were associated with cellular mechanisms responsible for glucose handling rather than the disease per se [49]. The patients involved in our study were on anti-diabetic agents such as gliclazide and metformin, medication shown to have epigenetic effects [50, 51]. It has been shown that metformin not only improves glucose metabolism but also exhibits anti-inflammatory activity via blockage of the PI3K-AKT pathway that reduced NF-κB translocation [52, 53]. In line with our results, anti-inflammatory cytokine levels in healthy adults, particularly IL-1Ra [54], IL-10 [55], and TGF-β [56] have been found to increase with age. This phenomenon was linked to the low grade inflammation state in the elderly, which often referred to as inflammaging and might be caused by age-dependent global hypomethylation [57]. The lack of correlation between age and *IL1RN*/IL-1Ra plasma levels in T2DM, regardless of the important role of aging, suggests the independent nature of influence of genetic factors and/or treatment on methylation status of *IL1RN* gene.

## Conclusion

To the best of our knowledge, the study provides the first evidence of hypomethylation of *IL1RN* and *NFKB1* genes which may contribute to the immunologic landscape of T2DM. The balance between IL-1β and IL-1Ra is a major determinant of the time course and severity of inflammatory diseases. However, it is questionable whether IL-1Ra is able to exhibit protective effect for the disease progression, since clinical evidence showed that IL-1Ra/IL-1β ratio of > 1000 (500 fold in our study) may inhibit inflammation [58]. We can speculate that increased release of IL-1Ra in combination with other anti-inflammatory mediators in the setting of diabetes is likely to contribute to the attenuating of inflammatory phenotype in treated patients. Although our findings highlight a role for *IL1RN* and *NFKB1* genes in disease pathogenesis, further studies are required to reveal the mechanisms by which hypomethylation of IL-1Ra and NF-κB1 (p50) coding genes affect the clinical course of T2DM patients.

## Supporting information

S1 Fig The ratio of IL-1Ra/IL-1β plasma levels in healthy controls (Healthy) and type 2 diabetes mellitus (T2DM) patients and separately in male/female subjects. Results are expressed as mean ± SEM. **P<0.01, ***P< 0.0001.
(TIFF)

S2 Fig Correlations analyses between (A) IL-1β and IL-1Ra plasma levels in healthy controls (Healthy) and type 2 diabetes mellitus (T2DM) patients; (B) Mean DNA methylation status of *IL1RN* gene/IL-1Ra plasma levels and age in healthy controls.
(TIF)

S3 Fig Correlation between LPS-stimulated production of IL-1Ra/IL-1β and fasting glucose levels in type 2 diabetes mellitus (T2DM) patients. LPS-stimulation index: calculated as the ratio of the cytokine production of LPS-stimulated cells to that of cells cultured without LPS.
(TIFF)

S4 Fig Stimulation of THP-1 cells with recombinant IL-1β (2.5ng/mL) and IL-1Ra (250ng/mL and 500ng/mL) in low (L-Glu) and high glucose (H-Glu) conditions: (A) Median fluorescence intensity (MFI) of CXCR1 (n = 6); (B) Percentage of pHrodo^™^ positive cells (n = 8); (C) Production of IL-10 (n = 7); (D) Production of TNF-α (n = 7). Results are expressed as mean ± SEM. *P< 0.05, **P< 0.001.
(TIFF)

S5 Fig Dynamic of *IL1RN* gene promoter methylation and IL-1Ra/IL-1β plasma levels in T2DM and healthy female subjects. (TIFF)

S1 Table Difference between DNA methylation levels of individual CpG loci located in *IL1RN* gene promoter. Results are expressed as mean ± SD. *P< 0.05.
(DOCX)
