# Supplementary figures and images for: Hypomethylation of IL1RN and NFKB1 genes is linked to the dysbalance in IL1β/IL-1Ra axis in female patients with type 2 diabetes mellitus
Source: PLoS One. 2020 May 29;15(5):e0233737. doi: 10.1371/journal.pone.0233737 (PMC7259508; doi:10.1371/journal.pone.0233737)

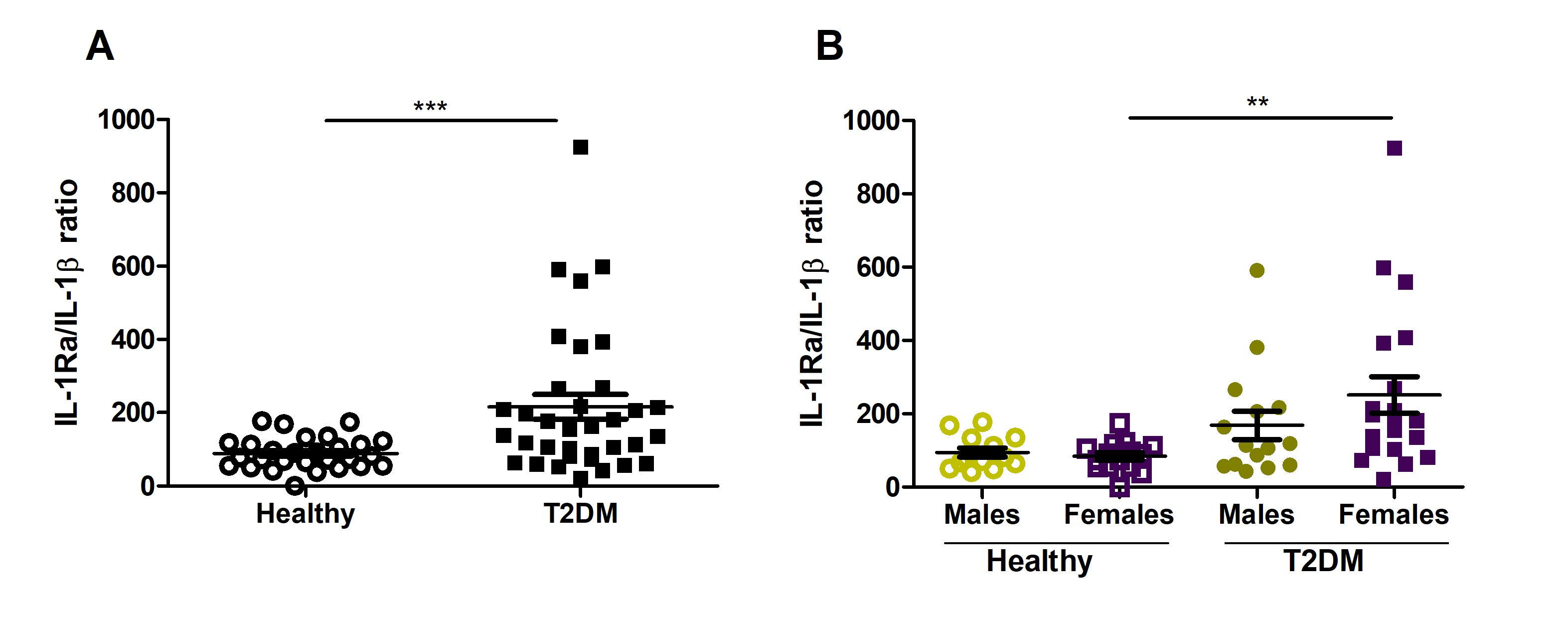

Supplement: S1 Fig — Results are expressed as mean ± SEM. **P<0.01, ***P< 0.0001. (TIFF) [file pone.0233737.s001.tiff]

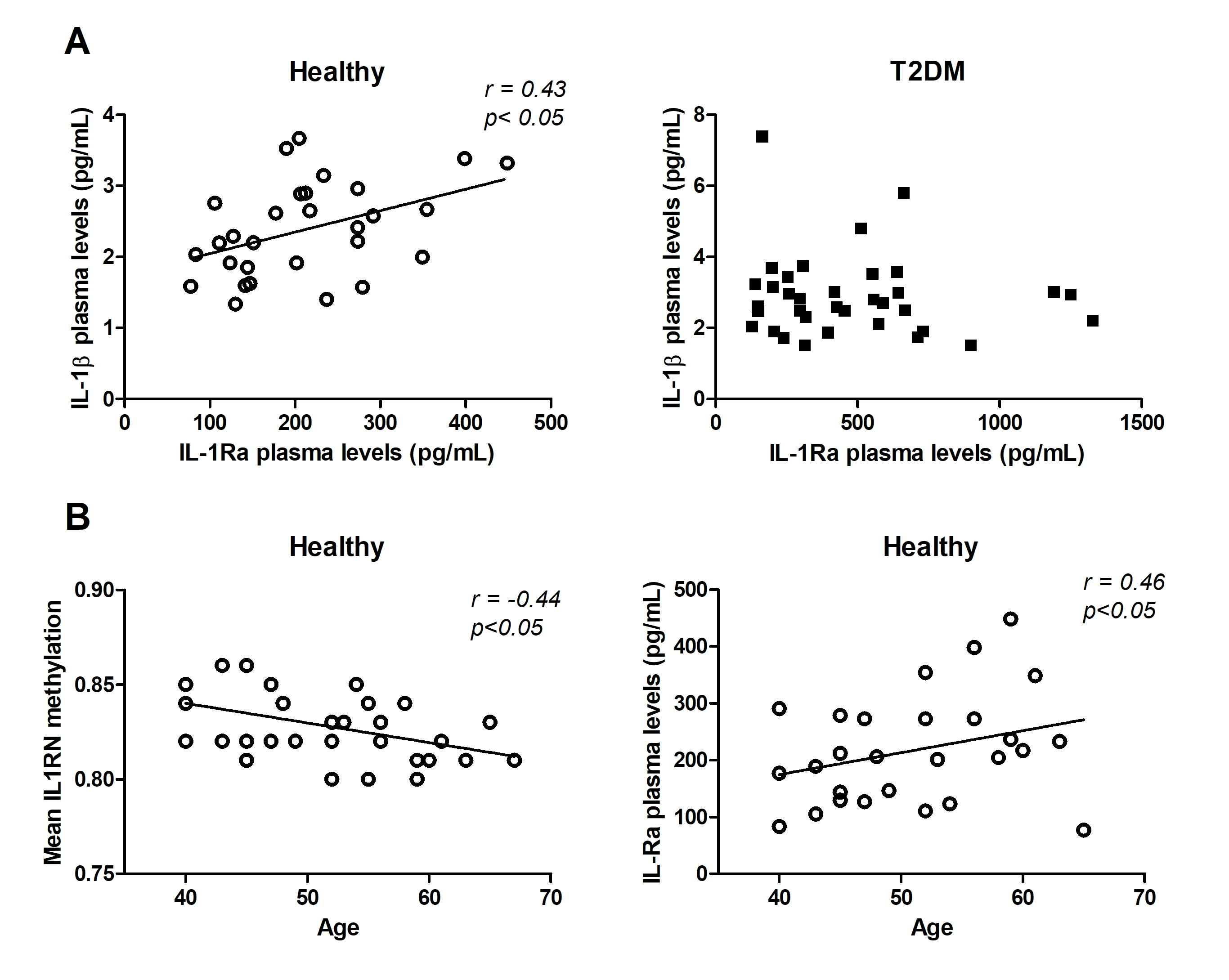

Supplement: S2 Fig — Correlations analyses between (A) IL-1β and IL-1Ra plasma levels in healthy controls (Healthy) and type 2 diabetes mellitus (T2DM) patients; (B) Mean DNA methylation status of IL1RN gene/IL-1Ra plasma levels and age in healthy controls. (TIF) [file pone.0233737.s002.tif]

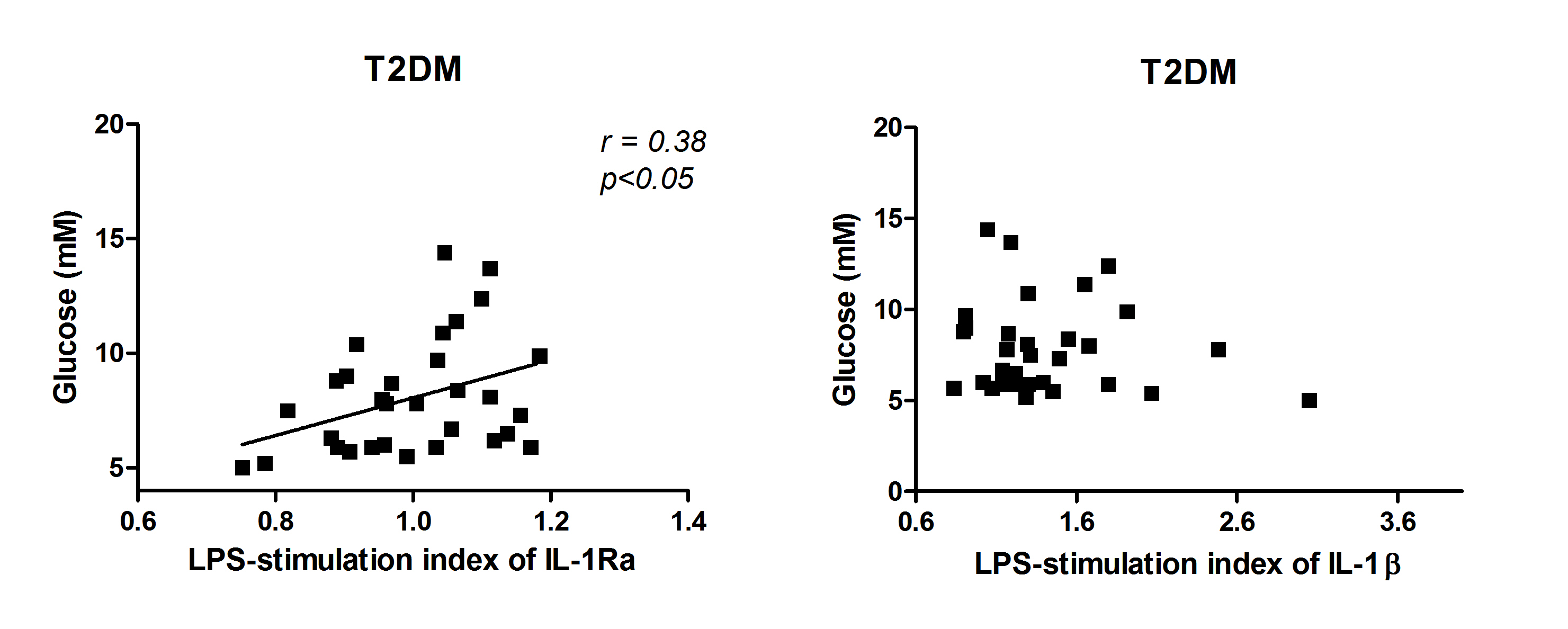

Supplement: S3 Fig — LPS-stimulation index: calculated as the ratio of the cytokine production of LPS-stimulated cells to that of cells cultured without LPS. (TIFF) [file pone.0233737.s003.tiff]

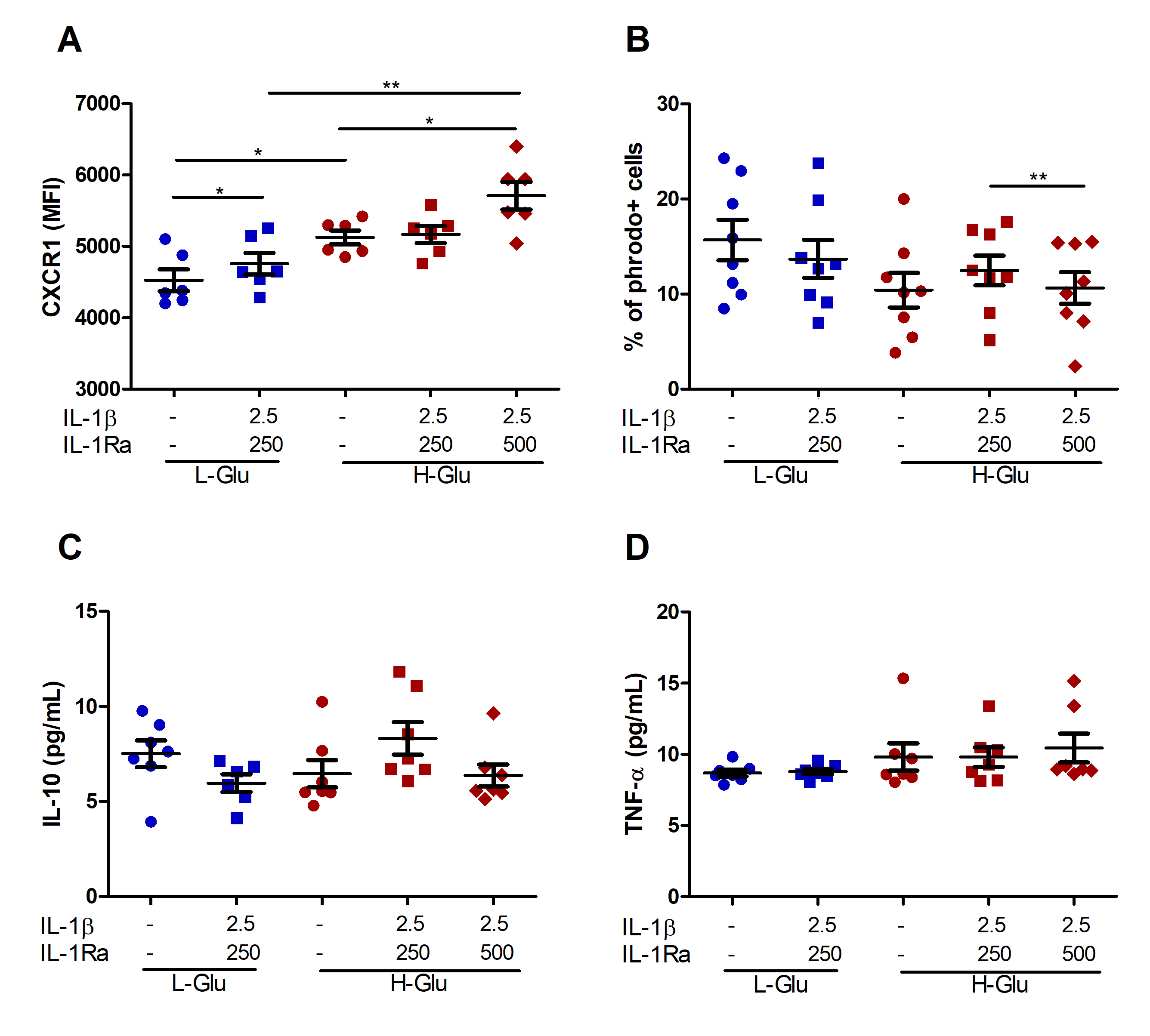

Supplement: S4 Fig — Stimulation of THP-1 cells with recombinant IL-1β (2.5ng/mL) and IL-1Ra (250ng/mL and 500ng/mL) in low (L-Glu) and high glucose (H-Glu) conditions: (A) Median fluorescence intensity (MFI) of CXCR1 (n = 6); (B) Percentage of pHrodo™ positive cells (n = 8); (C) Production of IL-10 (n = 7); (D) Production of TNF-α (n = 7). Results are expressed as mean ± SEM. *P< 0.05, **P< 0.001. (TIFF) [file pone.0233737.s004.tiff]

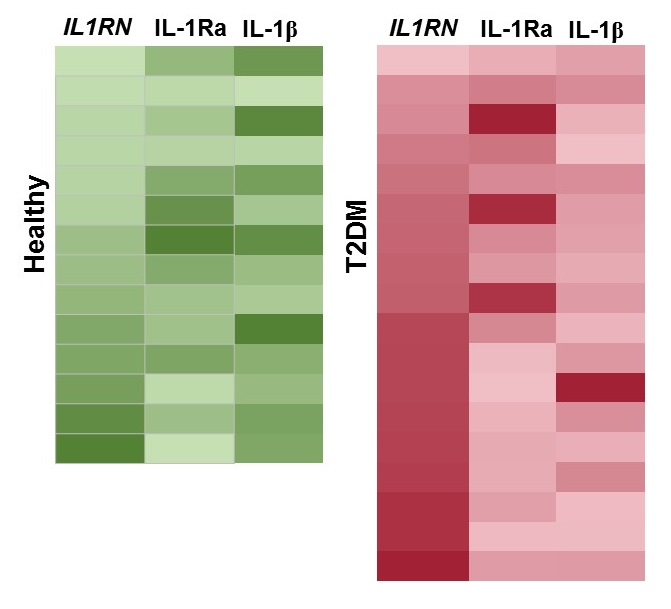

Supplement: S5 Fig — (TIFF) [file pone.0233737.s005.tiff]
