# Supplementary material for: Hypomethylation of IL1RN and NFKB1 genes is linked to the dysbalance in IL1β/IL-1Ra axis in female patients with type 2 diabetes mellitus
Source: PLoS One. 2020 May 29;15(5):e0233737. doi: 10.1371/journal.pone.0233737 (PMC7259508; doi:10.1371/journal.pone.0233737)
Supplement: S1 Table — Results are expressed as mean ± SD. *P< 0.05. (DOCX) [file pone.0233737.s006.docx]

|  | **Healthy** | **T2DM** |  |
| --- | --- | --- | --- |
| ***CpG position*** | ***Mean ± SD*** | | ***P value*** |
| CpG 41 | 0.8719 ± 0.04 | 0.8715 ± 0.042 | 0.9638 |
| CpG 95 | 0.8416 ± 0.051 | 0.8333 ± 0.055 | 0.5269 |
| CpG 97 | 0.9443 ± 0.063 | 0.9321 ± 0.081 | 0.7671 |
| CpG 126 | 0.8096 ± 0.063 | 0.8046 ± 0.081 | 0.781 |
| CpG 132 | 0.8074 ± 0.048 | 0.8026 ± 0.044 | 0.6697 |
| CpG 151 | 0.7410 ± 0.113 | 0.6925 ± 0.19 | 0.6967 |
| CpG 168 | 0.7327 ± 0.053 | 0.7381 ± 0.053 | 0.6812 |
| CpG 185 | 0.8517 ± 0.089 | 0.8279 ± 0.159 | 0.9731 |
| CpG 192 | 0.8355 ± 0.061 | 0.8088 ± 0.108 | 0.2866 |
| CpG 232 | 0.8616 ± 0.046 | 0.8480 ± 0.086 | 0.4356 |
| CpG 302 | 0.8384 ± 0.038 | 0.7903 ± 0.117 | ***0.022**** |
| CpG 317 | 0.8139 ± 0.079 | 0.8162 ± 0.047 | 0.8857 |
| CpG 343 | 0.9548 ± 0.064 | 0.9291 ± 0.1 | 0.7376 |
| CpG 359 | 0.7210 ± 0.062 | 0.7225 ± 0.08 | 0.9334 |
| CpG 376 | 0.6873 ± 0.067 | 0.7106 ± 0.081 | 0.2137 |
| CpG 383 | 0.9200 ± 0.04 | 0.8947 ± 0.052 | ***0.0338**** |
